# Development of a diagnostic test set to assess agreement in breast pathology: practical application of the Guidelines for Reporting Reliability and Agreement Studies (GRRAS)

**DOI:** 10.1186/1472-6874-13-3

**Published:** 2013-02-05

**Authors:** Natalia V Oster, Patricia A Carney, Kimberly H Allison, Donald L Weaver, Lisa M Reisch, Gary Longton, Tracy Onega, Margaret Pepe, Berta M Geller, Heidi D Nelson, Tyler R Ross, N AAnna Tosteson, Joann G Elmore

**Affiliations:** 1 Department of Medicine, University of Washington, Seattle, WA, USA; 2 Department of Family Medicine, Oregon Health and Science University, Portland, OR, USA; 3 Department of Pathology, Stanford University School of Medicine, Palo Alto, CA, USA; 4 Department of Pathology, University of Vermont and Vermont Cancer Center, Burlington, VT, USA; 5 Public Health Sciences Division, Fred Hutchinson Cancer Research Center, Seattle, WA, USA; 6 Norris Cotton Cancer Center and The Dartmouth Institute for Health Policy and Clinical Practice, Geisel School of Medicine at Dartmouth, Hanover, NH, USA; 7 Office of Health Promotion Research, University of Vermont, Burlington, VT, USA; 8 Department of Medical Informatics and Clinical Epidemiology, Oregon Health and Science University, Portland, OR, USA

**Keywords:** Reporting guidelines, Reliability of results, Agreement studies, Breast, Pathology, Diagnostic techniques

## Abstract

**Background:**

Diagnostic test sets are a valuable research tool that contributes importantly to the validity and reliability of studies that assess agreement in breast pathology. In order to fully understand the strengths and weaknesses of any agreement and reliability study, however, the methods should be fully reported. In this paper we provide a step-by-step description of the methods used to create four complex test sets for a study of diagnostic agreement among pathologists interpreting breast biopsy specimens. We use the newly developed Guidelines for Reporting Reliability and Agreement Studies (GRRAS) as a basis to report these methods.

**Methods:**

Breast tissue biopsies were selected from the National Cancer Institute-funded Breast Cancer Surveillance Consortium sites. We used a random sampling stratified according to woman’s age (40–49 vs. ≥50), parenchymal breast density (low vs. high) and interpretation of the original pathologist. A 3-member panel of expert breast pathologists first independently interpreted each case using five primary diagnostic categories (non-proliferative changes, proliferative changes without atypia, atypical ductal hyperplasia, ductal carcinoma in situ, and invasive carcinoma). When the experts did not unanimously agree on a case diagnosis a modified Delphi method was used to determine the reference standard consensus diagnosis. The final test cases were stratified and randomly assigned into one of four unique test sets.

**Conclusions:**

We found GRRAS recommendations to be very useful in reporting diagnostic test set development and recommend inclusion of two additional criteria: 1) characterizing the study population and 2) describing the methods for reference diagnosis, when applicable.

## Background

In 2011, the Guidelines for Reporting Reliability and Agreement Studies (GRRAS) were developed to outline and describe key methodological issues that should be addressed when reporting on reliability and agreement studies [1]. Clinicians’ interpretive reliability and diagnostic agreement are often evaluated using test sets. Yet, the methods used to develop these test sets are complex and multi-faceted. The development of test sets can lead to inherent biases, such as selecting high-quality cases only or selecting cases from unique clinical practices rather than random selection from large databases or populations [2-8]. To fully understand the strengths and weaknesses of any study that utilizes test set methodology, the development of the test set must be fully described and reported. The GRRAS recommendations provide a framework for doing so.

Most of the 15 GRRAS recommendations refer to the presentation of study methods and interpretation of results (Table 1). These include describing the diagnostic or measurement device of interest, specifying the rater population, and providing an in-depth description of the sampling methods and sample size for both the subject and rater populations.

**Table 1:** Current GRRAS Guidelines and Suggested Additions to GRRAS

	**Current Guidelines for Reporting Reliability and Agreement Studies (GRRAS) [**[1]**]**	**Suggested Additions to GRRAS**
TITLE AND ABSTRACT	1. Identify in title or abstract that interrater/intrarater reliability or agreement was investigated.	
INTRODUCTION	2. Name and describe the diagnostic or measurement device of interest explicitly.	
	3. Specify the subject population of interest.	Describe the database used to select the cases and the quality of that data.
	4. Specify the rater population of interest (if applicable).	
	5. Describe what is already known about reliability and agreement and provide a rationale for the study (if applicable).	
METHODS	6. Explain how the sample size was chosen. State the determined number of raters, subjects/objects, and replicate observations.	Describe the sampling method and the underlying population of both subjects and raters.
		7. Define the Reference Standard diagnosis.
	8. Describe the sampling method.	
	9. Describe the measurement/rating process (e.g. time interval between repeated measurements, availability of clinical information, blinding).	
	10. State whether measurements/ratings were conducted independently.	
	11. Describe the statistical analysis.	
RESULTS	12. State the actual number of raters and subjects/objects which were included and the number of replicate observations which were conducted.	
	13. Describe the sample characteristics of raters and subjects (e.g. training, experience).	
	14. Report estimates of reliability and agreement including measures of statistical uncertainty.	
DISCUSSION	15. Discuss the practical relevance of results.	
AUXILIARY MATERIAL	16. Provide detailed results if possible (e.g. online).	

In this paper, we use GRRAS recommendations to provide an overview of our methods to create four diagnostic test sets for a study assessing agreement among pathologists in the interpretation of breast biopsy specimens. The aims of the larger study include determining relationships between characteristics of patients and pathologists and diagnostic accuracy of biopsy specimens. In this paper, we focus on describing the sampling and random assignment methods (GRRAS criteria 1–13, Table 1) used to create the diagnostic test sets, as well as the demographics of the patient subjects whose breast biopsies were included in the test set cases. We also identify and discuss important additional criteria that would make GRRAS stronger and potentially even more relevant when test sets are employed. The statistical analysis and results of the inter-rater agreement of the test set cases will be reported elsewhere.

## Methods and materials

### IRB approval and consenting process

Biopsy specimens for the test set cases were identified and obtained from the Breast Cancer Surveillance Consortium (BCSC) registries in Vermont and New Hampshire [9-11]. The BCSC is a collaborative network of five geographically distinct mammography registries with linkages to breast pathology and/or tumor registries [10]. BCSC procedures are Health Insurance Portability and Accountability Act (HIPAA) compliant and all registries have a Federal Certificate of Confidentiality and other protection for the identities of research subjects and the physicians and facilities that contribute data to the BCSC [12]. Women enrolled in BCSC registries provided prior consent to BCSC investigators allowing their archived tissue samples to be used for research [10]. Thus, the research subjects did not need to be re-consented for the development of the test sets created for the current study.

Institutional Review Boards at the University of Washington, Dartmouth College, the University of Vermont, Fred Hutchinson Cancer Research Center, and Providence Health & Services of Oregon approved all test set study activities. A study-specific Certificate of Confidentiality (NCI 11–049) was also obtained to protect the study findings from forced disclosure of identifiable information.

### Test set case identification and selection

All biopsies used for the test set cases were performed between January 1, 2000 and December 31, 2008. Only excisional and core needle biopsies were used. Total mastectomy cases and fine needle aspiration specimens were excluded. Only one biopsy per woman was selected. When multiple biopsies were available from a single woman we randomly selected a biopsy within a hierarchical classification of cases with available clinical history. We prioritized biopsies in which the woman’s hormone therapy (HT) status at time of biopsy was known. If HT status was unknown, cases were selected by availability of information on family history. If both HT and family history were unknown cases were selected by known race.

All women with a previous history of breast cancer were excluded. A family history of breast cancer was defined as having a first degree relative (i.e., mother, daughter or sister) with a breast cancer diagnosis. Breast cancer history was assessed through a yes/no response to the following questions, depending on the BCSC site, on a risk factor questionnaire: “Have you ever been diagnosed with breast cancer?” or “Has the patient ever had breast cancer?”. A total of 19,498 biopsies obtained from 13,677 distinct women met our eligibility requirements.

Test set cases were selected using random stratified sampling based on the age of the woman (40–49 vs. ≥50), breast density (low vs. high), and the final diagnostic interpretation of the original BCSC contributing pathologist who reviewed the woman’s biopsy for clinical treatment and management. Contributing BCSC pathologists include a variety of practice settings ranging from private practices in small hospitals to large University-affiliated academic practices in tertiary medical centers. For all potential test set cases, we categorized the diagnostic interpretations of the BCSC pathologist into one of five diagnostic classifications (non-proliferative changes, proliferative changes without atypia, atypical ductal hyperplasia (ADH), ductal carcinoma in situ (DCIS), and invasive breast carcinoma). This resulted in 20 possible combinations of test set cases (5 diagnostic categories x 2 age groups x 2 breast density groups = 20 combinations). Low versus high breast density was defined as ≤ 50% fibroglandular (BI-RADS categories 1 and 2) or ≥ 51% fibroglandular (BI-RADS categories 3 and 4), respectively. Information on breast density was obtained from mammography exams in the three years prior to biopsy. We used data from the most proximal mammogram for women with multiple mammograms within the three year period.

We oversampled cases of ADH and DCIS compared to national estimates of biopsy outcomes in the U.S. [13] to increase statistical confidence and raw rates of inter-observer variation in areas of breast pathology that may be more challenging to interpret, are lower frequency diagnoses, and where disagreements would affect treatment. Population-based adjustments according to disease prevalence will be made during statistical analysis.

Previous studies have shown misclassification rates among pathologists of over 50% for ADH and 17% for DCIS [14]. Women in their 40s and women with dense breast tissue were also oversampled because age and breast density are known risk factors for both benign breast disease and breast cancer [15], and because our *a priori* hypotheses include that there is more diagnostic variability in biopsies from women aged 40–49 years and women with dense breast tissue. By design, half of the test set cases were from women aged 40–49 at the time of the biopsy and half were from women aged 50 and older, with no upper age limit. Also by design, half of the cases were from women with high-density breast tissue and half were from women with low-density breast tissue.

**Figure 1:**
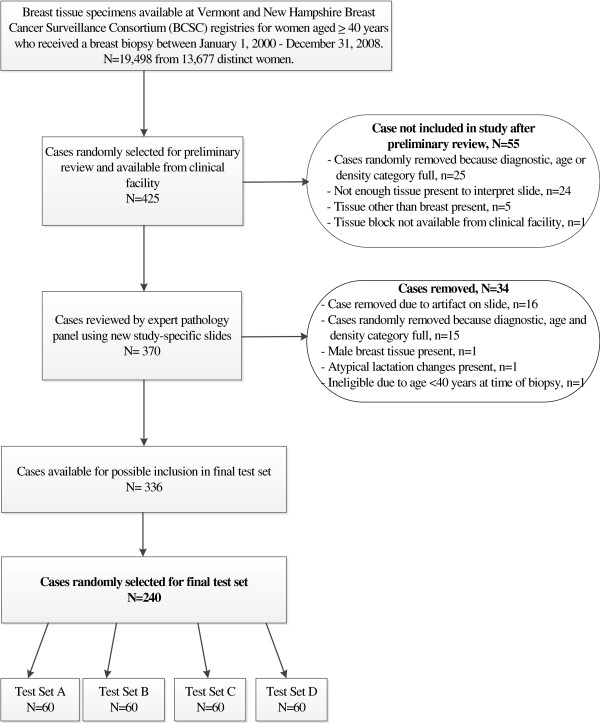
Flow chart describing test set development.

A listing of candidate cases was randomly identified from the Vermont and New Hampshire BCSC registries (data not shown). Tissue blocks and original slides were requested from clinical facilities in Vermont (n=8) and New Hampshire (n=8) for review by our expert pathology panel. If a facility did not send the material after three written requests and two phone requests, we removed that case from our selection and requested the next case on the list within the 20 selection categories until we met our target accrual of 425 cases (Figure 1). Not all cases were available at the time of request. Reasons for a case being unavailable included insufficient residual tissue in paraffin blocks, the facility required additional consent procedures, or the tissue had already been discarded.

### Initial review of biopsy material

An expert pathology panel comprised of three internationally recognized breast pathologists reviewed all selected cases. One expert panel member conducted an initial assessment of all original slides associated with the biopsy received from the clinical facility. The woman’s age and biopsy type (core needle or excisional) were the only clinical history provided for each case. This expert was blinded to the original diagnosis made by the contributing pathologist. After the initial review was complete, new study-specific glass slides were created from each case’s appropriate paraffin embedded tissue block(s) to ensure consistent staining and image quality. The newly created slides were used for the full expert panel review.

### Standardized data collection

We developed a standardized histology data collection form, called the Breast Pathology Assessment Tool Hierarchy (B-PATH) form, which the expert pathologists used to record detailed diagnostic information about each case during their review. The B-PATH form included the same five diagnostic categories that were used for case selection (non-proliferative changes, proliferative changes without atypia, ADH, DCIS, and invasive breast carcinoma). The expert pathologists were asked to indicate if the case was borderline between two diagnostic categories and whether they would have requested a second diagnostic opinion for the case had they seen it in clinical practice. Finally, the B-PATH form asked pathologists to rate the level of diagnostic difficulty and their level of confidence in the assessment of each case using a 6-point Likert scale, with 1 representing “very easy” or “very confident” and 6 representing “very challenging” or “not confident”, respectively.

### Independent and consensus review by expert panel

The 3-member expert pathologist panel (including the expert who conducted the initial review of the original tissue slides) performed blinded independent assessments on each slide using the standardized B-PATH form.

We used a modified Delphi approach [16] to establish the final reference standard diagnosis for each slide. This involved compiling the independent reviews for each case, providing the three experts with their initial interpretation of the slides, followed by a facilitated discussion of features and diagnostic criteria of areas where disagreement among the experts occurred during a re-review of the slide(s) at a multi-headed microscope. The facilitated discussion continued until a final consensus was reached among all three expert pathologists on the case interpretation. When more than one slide was available for a case, the panel selected the slide that was most representative of the diagnosis.

### Sample size calculations and random assignment of cases into four diagnostic test sets

The number of cases per test set and the number of participating study pathologists were chosen to provide sufficient power to address the study aims. Using conservative assumptions about diagnostic variability among pathologists, [14] we determined that 60 cases per test set of glass slides interpreted by 100 participating pathologists would, for example, yield 90% power to detect an effect of patient age (40–49 vs. ≥50 years) on a misclassification rate difference as small as 4.8% when interpreting cases with atypia and DCIS.

After the three expert pathologists reached final diagnostic consensus, and each case had been mapped into one of five primary diagnostic categories (Table 2), 240 unique patient cases were randomly selected from a total of 336 cases reviewed by the expert panel (Figure 1). Selection was performed within cells defined by three stratification factors in order to obtain the desired distribution of cases across factors. These included 1) case diagnosis (using the five diagnostic categories on the B-PATH data collection form), 2) patient age (40–49 vs. ≥50), and 3) breast density (low vs. high), respectively. A permuted block randomization method with block size of four was used to assign cases to the four test sets. Blocks were defined within strata by similarity of case difficulty score (i.e., the mean Likert rating on the difficulty level assigned to each case by the three expert pathologists). For strata in which the cell total was not evenly divisible by four, random permutations of the relevant sets were assigned to the remaining partial block. Four final test sets were developed, each of which contained 60 unique patient cases.

**Table 2:** Diagnostic Breast Pathology Assessment Tool Hierarchy (B-PATH) mapping categories for test set cases

**Primary clinical diagnostic category**	**All diagnoses included in primary clinical diagnostic categories**
I. Non-Proliferative*	Non-Proliferative only
	Fibroadenoma
II. Proliferative changes without atypia	Usual ductal hyperplasia
	Columnar cell hyperplasia/columnar cell changes
	Sclerosing adenosis
	Radial Scar/complex sclerosing lesion
	Flat epithelial atypia
	Intraductal papilloma w/o atypia
III. Atypical ductal hyperplasia (ADH)	Atypical ductal hyperplasia
	Intraductal papilloma with atypia
IV. Ductal carcinoma in situ (DCIS)	Ductal carcinoma in situ
V. Invasive breast cancer	Invasive (ductal or lobular or other special type)

*For development of the test set, cases were categorized according to the highest ductal proliferative or malignant lesion present on the slide. When only lobular carcinoma in situ (LCIS) or atypical lobular hyperplasia (ALH) was present (n=2), we categorized cases as non-proliferative.

We aimed to create a test set of slides that, as closely as possible, mirrored the quality and variety of cases observed in everyday clinical practice. We recognize that there is a wide range of diagnostic quality of source material; our statistical sampling and selection methods were designed to eliminate or minimize selection bias. Cases were deemed ineligible only when there was slide preparation artifact or insufficient tissue present to interpret the slide (n=40), tissue other than breast tissue was present on the slide due to a contributing facility supplying an incorrect block (n=5), the tissue block was unavailable (n=1), male breast tissue was present (n=1), or atypical lactation changes were present (n=1). The final set of eligible cases selected may not be considered necessarily easy or difficult to interpret, or ideal for teaching purposes because the selection process was designed to circumvent the type of selection bias that may exist in typical continuing medical educational conferences and courses.

The results of the inter-rater agreement of the test sets and a description of how the test sets will be used to assess agreement in pathologists’ diagnostic interpretation of breast tissue will be reported elsewhere. In brief, approximately 200 study pathologists will be invited to independently review the test set cases and provide their diagnostic interpretation. These interpretations will be compared to the reference standard diagnoses as determined by the expert pathology panel and to the interpretations of other pathologists in the study.

## Discussion

In this paper we provide an example of how GRRAS recommendations can be applied to developing and reporting the methods used to create diagnostic test sets to assess interpretive reliability and diagnostic agreement. Previous studies have described the poor quality of research reporting [17-19] but, encouragingly, research has also shown that the development and use of formalized guidelines improves the quality of reporting [20]. The methods that are used to develop test sets contribute importantly to the validity of study findings. Thus, providing a clear description of methodological details and standardized terminology [21] is critical in assisting readers to assess the strengths and shortcomings of the study findings, especially as they relate to implications for clinical care.

Because the GRRAS recommendations are new, Kottner et al. [1] invited researchers to provide feedback on whether use of the guidelines improves reporting for studies of reliability and agreement, and to suggest updates for the guidelines themselves. Our investigative team found GRRAS very helpful in describing the complex methods of developing a diagnostic breast pathology test set. In addition to the current GRRAS recommendations, we suggest the inclusion of additional guidelines for test set development that may be applicable to other studies. First, we suggest that data sources for patient populations be well characterized, both in terms of geographic and health system features. This is important because if patients are from a small geographic area (e.g., a single clinic or hospital) they will likely be different from those identified from a large geographic sample or dataset and as such will affect the generalizability of the study results. Second, if accuracy is being investigated, researchers should describe the methods used to define the reference diagnosis or gold standard. The importance of documenting this is widely recognized in guidelines for reporting diagnostic accuracy studies such as STARD [22]. While some studies of inter-rater reliability are designed to evaluate agreement, it is often more clinically useful to understand accuracy. Studies can be designed using external tests or additional clinical follow-up to define a reference standard diagnosis. For example, two years of follow-up can be used to determine if breast cancer develops in studies of variability of radiologists’ interpretation of mammography, [23] and echocardiography can be used as the reference standard to assess the diagnostic accuracy of physicians identifying cardiac lesion type and severity by auscultation [24].

Breast cancer diagnosis and treatment relies on pathologists’ interpretation of breast biopsy specimens. However, studies investigating levels of interpretive agreement when diagnosing breast cancer and precursor lesions are limited [8,14]. The substantial clinical disagreement in pathological interpretation of borderline cases raises concern about over or under treatment of women with precursor lesions such as ADH and DCIS. In addition, misclassifications alter the study of patient outcomes associated with effective treatment. The use of carefully developed test sets provides an opportunity to improve our understanding of interpretive variation and its underlying causes.

### Strengths and limitations

Studies of observer reliability and accuracy often rely on test sets. Yet, the use of test sets, no matter how carefully developed, opens the door to limitations shared by these studies, including ours. First, the use of test sets may cause clinicians to modify their opinion to reflect what they believe is the “right” response [25,26]. Second, performance on test sets may not reflect performance in clinical practice [25,27]. An ideal study design might include embedding test cases into actual clinical practice while keeping the interpreting clinician blinded to test cases. However, it is logistically impractical to keep pathologists blinded to test cases in studies that involve large numbers of physicians from multiple geographic locations and a large number of test cases. Third, as described by Kundel et al. [28] accuracy in many studies is only implied with the assumption that when readers agree they must be correct. However, readers may all agree and also be wrong. There is not a perfect method to define a reference standard, but studies have found that the use of consensus ratings by experts show more accurate estimates of case diagnosis and outcomes compared to non-consensus trials, even when raters’ bias is considered [29]. By requiring complete consensus of our experts on their diagnostic interpretation of all of our test set cases, we expect to achieve the most rigorous reference diagnosis possible for each test set case in this study.

Our meticulously developed test set used a random case selection process within specific diagnostic categories and had a large sample size, allowing our study design to modify some of the problems inherent in previously reported observer variability studies. Test sets are typically selected with a focus on difficult cases, narrowly selected diagnoses or cases handpicked for unique attributes or high quality. By contrast, our randomly selected test set excluded cases only when there was insufficient diagnostic tissue on the slide or incorrect source tissue blocks were supplied. For this reason, the test sets included cases that some clinicians might interpret as having borderline diagnoses and cases that may not typically be considered ideal for a teaching or training set, but that more accurately reflect the real-world spectrum of findings within each diagnostic category.

A unique strength of our study is that the case selection methods will allow us to eventually combine our test set interpretive findings with data on the diagnostic distributions of breast biopsy cases from all National Cancer Institute BCSC sites, which are representative of the U.S. population [10,30]. Because the study cases oversampled ADH and DCIS cases we will carefully model the data taking into consideration the prevalence of each diagnostic category in the U.S., based on previously published BCSC data [13] and new data that we are collecting from the BCSC. Study findings will be combined with other U.S. data to provide population estimates on the impact that alternative breast pathology reading practices may have on health and resource use among women undergoing breast biopsies.

## Conclusions

The use of test sets is a valuable research tool that contributes importantly to the reliability and face validity in studies on agreement in diagnosis of biopsy specimens. The methods used to develop these test sets, however, are complex and multi-faceted and contribute to the accuracy of study results. The intent of GRRAS is to provide a framework to fully describe the methods by which a score, rating, or measurement has been determined [1] so that practitioners and researchers alike can weigh this information along with the study findings. Formalized guidance in reporting is welcomed by many researchers, and the use of guidelines has been found to improve the reporting of research results. We found GRRAS recommendations to be helpful, and encourage their use along with our suggested additions to GRRAS, characterizing the study population and describing the methods used to define the reference diagnosis, when applicable.

## Competing interests

In the past five years none of the authors have received reimbursements, fees, funding, or salary from an organization that may in any way gain or lose financially from the publication of this manuscript, either now or in the future. No financial organizations are financing this manuscript (including the article-processing charge). No authors hold stocks or shares in an organization that may in any way gain or lose financially from the publication of this manuscript, either now or in the future. None of the authors hold or are currently applying for any patents relating to the content of the manuscript. We have not received reimbursements, fees, funding, or salary from an organization that holds or has applied for patents relating to the content of the manuscript. We have no other financial competing interests to disclose. None of the authors have non-financial competing interests (political, personal, religious, ideological, academic, intellectual, commercial or any other) to declare in relation to this manuscript.

## Authors’ contributions

NVO: Participated in study design and implementation. Drafted manuscript. PAC: A key participant in the study design and manuscript preparation. Developed and facilitated the modified Delphi method during the expert panel’s consensus meetings which were used to determine the reference standard consensus diagnosis for test set cases. KHA: Participated in study design and conception. Edited manuscript. Key participant in diagnostic agreement discussions during expert consensus meetings. DLW: Key participant in expert consensus meeting and overall study design. Edited manuscript. LR: Participated in the study design and implementation. Facilitated diagnostic consensus meetings. Participated in drafting of manuscript. GL: Performed sample size calculations, performed random statistical assignment of all biopsy cases into four diagnostic test sets and provided oversight on statistical issues during development of the test set. TO: Participated in design of the study, manuscript preparation and editing. MP: Key participant in the statistical design of the study. Provided oversight on statistical analysis during study development and implementation. BMG: Participated in study design and conception. Co-facilitated the modified Delphi method during the expert panel’s consensus meetings. Edited manuscript. HN: Study design and conception, manuscript preparation. TR: Performed statistical analysis to identify and select test set cases from the BCSC. AT: Key participant in study conception and design. JGE: Overall study design, conception and implementation. Key participant in manuscript writing and editing. All authors read and approved the final manuscript.

## Pre-publication history

The pre-publication history for this paper can be accessed here:

http://www.biomedcentral.com/1472-6874/13/3/prepub

## References

[B1] Kottner J Audige L Brorson S Donner A Gajewski BJ Hrobjartsson A Roberts C Shoukri M Streiner DL Guidelines for reporting reliability and agreement studies (GRRAS) were proposed J Clin Epidemiol 2011 64 1 96 106 10.1016/j.jclinepi.2010.03.002 21130355

[B2] Schnitt SJ Connolly JL Tavassoli FA Fechner RE Kempson RL Gelman R Page DL Interobserver reproducibility in the diagnosis of ductal proliferative breast lesions using standardized criteria Am J Surg Pathol 1992 16 12 1133 1143 10.1097/00000478-199212000-00001 1463092

[B3] Rosai J Borderline epithelial lesions of the breast Am J Surg Pathol 1991 15 3 209 221 10.1097/00000478-199103000-00001 1847606

[B4] Petralia G Bonello L Summers P Preda L Malasevschi A Raimondi S Di Filippi R Locatelli M Curigliano G Renne G Intraobserver and interobserver variability in the calculation of apparent diffusion coefficient (ADC) from diffusion-weighted magnetic resonance imaging (DW-MRI) of breast tumours Radiol Med 2011 116 3 466 476 10.1007/s11547-011-0616-z 21225368

[B5] Imamura T Isomoto I Sueyoshi E Yano H Uga T Abe K Hayashi T Honda S Yamaguchi T Uetani M Diagnostic performance of ADC for Non-mass-like breast lesions on MR imaging Magn Reson Med Sci 2010 9 4 217 225 10.2463/mrms.9.217 21187691

[B6] Darvishian F Singh B Simsir A Ye W Cangiarella JF Atypia on breast core needle biopsies: reproducibility and significance Ann Clin Lab Sci 2009 39 3 270 276 19667411

[B7] Haupt B Schwartz MR Xu Q Ro JY Columnar cell lesions: a consensus study among pathology trainees Hum Pathol 2010 41 6 895 901 10.1016/j.humpath.2009.12.003 20233620

[B8] Wells WA Carney PA Eliassen MS Tosteson AN Greenberg ER Statewide study of diagnostic agreement in breast pathology JNCI 1998 90 2 142 145 10.1093/jnci/90.2.142 9450574

[B9] Weaver DL Vacek PM Skelly JM Geller BM Predicting biopsy outcome after mammography: what is the likelihood the patient has invasive or in situ breast cancer? Ann Surg Oncol 2005 12 8 660 673 10.1245/ASO.2005.09.008 15968496

[B10] Breast cancer surveillance consortium 2011 Available at: http://breastscreening.cancer.gov. Accessed June 1, 2011

[B11] Carney PA Poplack SP Wells WA Littenberg B The New hampshire mammography network: the development and design of a population-based registry Am J Roentgenol 1996 167 2 367 372 10.2214/ajr.167.2.8686606 8686606

[B12] Carney PA Geller BM Moffett H Ganger M Sewell M Barlow WE Stalnaker N Taplin SH Sisk C Ernster VL Current medicolegal and confidentiality issues in large, multicenter research programs Am J Epidemiol 2000 152 4 371 378 10.1093/aje/152.4.371 10968382

[B13] Weaver DL Rosenberg RD Barlow WE Ichikawa L Carney PA Kerlikowske K Buist DS Geller BM Key CR Maygarden SJ Pathologic findings from the breast cancer surveillance consortium: population-based outcomes in women undergoing biopsy after screening mammography Cancer 2006 106 4 732 742 10.1002/cncr.21652 16411214

[B14] Collins LC Connolly JL Page DL Goulart RA Pisano ED Fajardo LL Berg WA Caudry DJ McNeil BJ Schnitt SJ Diagnostic agreement in the evaluation of image-guided breast core needle biopsies: results from a randomized clinical trial Amer J Surg Path 2004 28 1 126 131 10.1097/00000478-200401000-00015 14707874

[B15] Ginsburg OM Martin LJ Boyd NF Mammographic density, lobular involution, and risk of breast cancer Brit J Cancer 2008 99 9 1369 1374 10.1038/sj.bjc.6604635 18781174 PMC2579686

[B16] Helmer-Hirschberg O The systematic Use of expert judgment in operations research 2012 Available at: http://www.rand.org/pubs/papers/P2795.html. Accessed August

[B17] Pocock SJ Collier TJ Dandreo KJ de Stavola BL Goldman MB Kalish LA Kasten LE McCormack VA Issues in the reporting of epidemiological studies: a survey of recent practice BMJ 2004 329 7471 883 10.1136/bmj.38250.571088.55 15469946 PMC523109

[B18] Honest H Khan KS Reporting of measures of accuracy in systematic reviews of diagnostic literature BMC Health Serv Res 2002 2 4 10.1186/1472-6963-2-4 11884248 PMC100326

[B19] Smidt N Rutjes AW van der Windt DA Ostelo RW Reitsma JB Bossuyt PM Bouter LM de Vet HC Quality of reporting of diagnostic accuracy studies Radiology 2005 235 2 347 353 10.1148/radiol.2352040507 15770041

[B20] Smidt N Rutjes AW van der Windt DA Ostelo RW Bossuyt PM Reitsma JB Bouter LM de Vet HC The quality of diagnostic accuracy studies since the STARD statement: has it improved? Neurology 2006 67 5 792 797 10.1212/01.wnl.0000238386.41398.30 16966539

[B21] National Health Service Breast Screening Programme Quality assurance guidelines for breast pathology services 2011 2 Available at: http://www.cancerscreening.nhs.uk/breastscreen/publications/nhsbsp02.pdf. Accessed December 2012

[B22] Bossuyt PM Reitsma JB Bruns DE Gatsonis CA Glasziou PP Irwig LM Moher D Rennie D de Vet HC Lijmer JG The STARD statement for reporting studies of diagnostic accuracy: explanation and elaboration Ann Intern Med 2003 138 1 W1 W12 12513067 10.7326/0003-4819-138-1-200301070-00012-w1

[B23] Elmore JG Wells CK Lee CH Howard DH Feinstein AR Variability in radiologists’ interpretations of mammograms N Engl J Med 1994 331 22 1493 1499 10.1056/NEJM199412013312206 7969300

[B24] Sztajzel JM Picard-Kossovsky M Lerch R Vuille C Sarasin FP Accuracy of cardiac auscultation in the era of doppler-echocardiography: a comparison between cardiologists and internists Int J Cardiol 2010 138 3 308 310 10.1016/j.ijcard.2008.06.066 18762344

[B25] Egglin TK Feinstein AR Context bias. A problem in diagnostic radiology JAMA 1996 276 21 1752 1755 10.1001/jama.1996.03540210060035 8940325

[B26] Bankier AA Levine D Halpern EF Kressel HY Consensus interpretation in imaging research: is there a better way? Radiology 2010 257 1 14 17 10.1148/radiol.10100252 20851935

[B27] Gur D Bandos AI Cohen CS Hakim CM Hardesty LA Ganott MA Perrin RL Poller WR Shah R Sumkin JH The “laboratory” effect: comparing radiologists’ performance and variability during prospective clinical and laboratory mammography interpretations Radiology 2008 249 1 47 53 10.1148/radiol.2491072025 18682584 PMC2607194

[B28] Kundel HL Polansky M Measurement of observer agreement Radiology 2003 228 2 303 308 10.1148/radiol.2282011860 12819342

[B29] Weller SC Mann NC Assessing rater performance without a “gold standard” using consensus theory Med Decis Making 1997 17 1 71 79 10.1177/0272989X9701700108 8994153

[B30] Ballard-Barbash R Taplin SH Yankaskas BC Ernster VL Rosenberg RD Carney PA Barlow WE Geller BM Kerlikowske K Edwards BK Breast cancer surveillance consortium: a national mammography screening and outcomes database Am J Roentgenol 1997 169 4 1001 1008 10.2214/ajr.169.4.9308451 9308451

